# *catena*-Poly[zinc(II)-μ-aqua-κ^2^ *O*:*O*-bis­(μ-quinoline-4-carboxyl­ato-κ^2^ *O*:*O*′)]

**DOI:** 10.1107/S1600536809025392

**Published:** 2009-07-04

**Authors:** Jin-Xi Chen, Wei-Wei Meng

**Affiliations:** a School of Chemistry and Chemical Engineering, Southeast University, Nanjing 211189, People’s Republic of China

## Abstract

The asymmetric unit of the title complex, [Zn(C_10_H_6_NO_2_)_2_(H_2_O)]_*n*_, consists of one quinoline-4-carboxyl­ate anion, half of a Zn^2+^ cation and half of a coordinated water mol­ecule. The cation and the water O atom have crystallographically imposed inversion and twofold rotation symmetry, respectively. The metal centre displays an elongated ZnO_6_ octa­hedral coordination geometry provided by the O atoms of four anions at the equatorial plane and two axial water mol­ecules. Each anion and water mol­ecule act as bridges between Zn^II^ cations, forming a polymeric chain parallel to [001]. The chains are further linked into a three-dimensional framework through O—H⋯N hydrogen bonds.

## Related literature

For the coordination chemistry of transition metal complexes with quinoline-4-carboxyl­ate, see: Bu *et al.* (2004 ▶, 2005 ▶); Xiong *et al.* (2000 ▶); Chen *et al.* (2002 ▶).
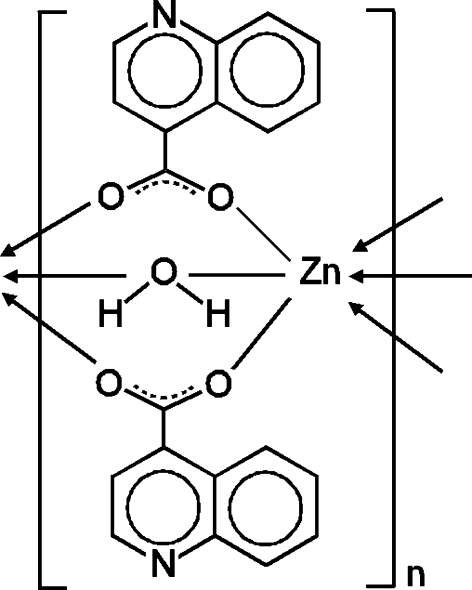


## Experimental

### 

#### Crystal data

[Zn(C_10_H_6_NO_2_)_2_(H_2_O)]
*M* *_r_* = 427.72
Monoclinic, 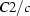

*a* = 14.929 (2) Å
*b* = 14.4025 (13) Å
*c* = 7.5428 (11) Å
β = 91.961 (6)°
*V* = 1620.8 (4) Å^3^
*Z* = 4
Mo *K*α radiation
μ = 1.56 mm^−1^
*T* = 293 K
0.30 × 0.30 × 0.20 mm

#### Data collection

Rigaku SCXmini diffractometer
Absorption correction: multi-scan (*CrystalClear*; Rigaku/MSC, 2005 ▶) *T*_min_ = 0.635, *T*_max_ = 0.732
5552 measured reflections
1831 independent reflections
1741 reflections with *I* > 2σ(*I*)
*R*_int_ = 0.030

#### Refinement

*R*[*F* ^2^ > 2σ(*F* ^2^)] = 0.026
*wR*(*F* ^2^) = 0.069
*S* = 1.09
1831 reflections
129 parameters
H-atom parameters constrained
Δρ_max_ = 0.38 e Å^−3^
Δρ_min_ = −0.82 e Å^−3^

### 

Data collection: *CrystalClear* (Rigaku/MSC, 2005 ▶); cell refinement: *CrystalClear*; data reduction: *CrystalClear*; program(s) used to solve structure: *SHELXS97* (Sheldrick, 2008 ▶); program(s) used to refine structure: *SHELXL97* (Sheldrick, 2008 ▶); molecular graphics: *SHELXTL* (Sheldrick, 2008 ▶); software used to prepare material for publication: *SHELXTL*.

## Supplementary Material

Crystal structure: contains datablocks I, global. DOI: 10.1107/S1600536809025392/rz2342sup1.cif

Structure factors: contains datablocks I. DOI: 10.1107/S1600536809025392/rz2342Isup2.hkl

Additional supplementary materials:  crystallographic information; 3D view; checkCIF report

## Figures and Tables

**Table 1:** Hydrogen-bond geometry (Å, °)

*D*—H⋯*A*	*D*—H	H⋯*A*	*D*⋯*A*	*D*—H⋯*A*
O3—H7⋯N1^i^	0.90	1.89	2.7920 (15)	174

Symmetry code: (i) 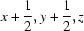.

**Fig. 1.**
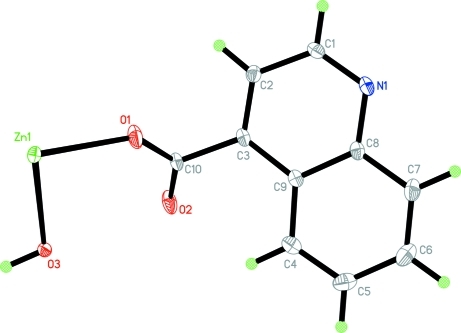
The asymmetric unit of the title compound, with displacement ellipsoids drawn at the 30% probability level.

**Fig. 2.**
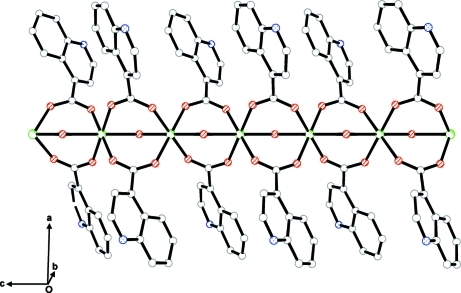
Partial crystal packing of the title compound, showing the polymeric chain parallel to [001]. Hydrogen atoms are omitted for clarity.

**Fig. 3.**
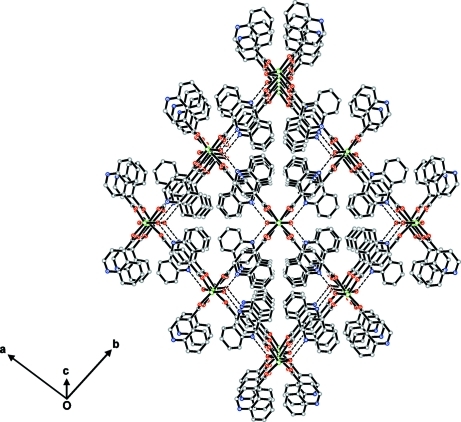
Crystal packing of the title compound viewed along the c axis. N—H···O hydrogen bonds are shown as dashed lines. Hydrogen atoms not involved in hydrogen bonding are omitted for clarity.

## supplementary crystallographic information

### Comment

In recent years, new coordination compounds based on transition metals and quinoline-4-carboxylic acid have attracted much attention because of the role of non-covalent supramolecular interactions such as hydrogen bonding or π-π conjugate effect (Bu *et al.* 2005). However, the use of quinoline-4-carboxylic acid for the construction of metal-organic frameworks has not been well documented yet (Bu *et al.*, 2004; Xiong *et al.*, 2000; Chen *et al.*, 2002).

The asymmetric unit of the title complex polymer (Fig. 1) consists of one quinoline-4-carboxylate anion, half of a zinc(II) cation and half of a coordinated water molecule. The cation and the water oxygen atom have crystallographically imposed inversion and twofold rotation symmetry, respectively. The geometry around the zinc(II) metal centre can be best described as elongated octahedral, with four oxygen atoms from four independent quinoline-4-carboxylate anions at the equatorial plane and two oxygen atoms from two H_2_O molecules at the axial position. Each quinoline-4-carboxylate anion adopts an *O,O'*-bidentate bridging mode. Adjacent zinc(II) cations are bridged by the quinoline-4-carboxylate ligands and water molecules, forming a chain parallel to [001] (Fig. 2). The chains are further linked into a three-dimensional network (Fig. 3) by O—H···N hydrogen bonds (Table 1).

### Experimental

The title compound was synthesized by the solvothermal reaction of Zn(NO_3_)_2_.6H_2_O (0.2 mmol, 0.0595 g), 4-quinolinecarboxylic acid (0.6 mmol, 0.1039 g) and C_2_H_5_OH/H_2_O (4:1 v/v; 5 ml) in a Teflon-lined autoclave at 180°C for 3 days. After the reaction autoclave was slowly cooled to room temperature for 24 h, light yellow block single crystals suitable for X-ray diffraction analysis were obtained, isolated by filtration and washed with water.

### Refinement

All H atoms were fixed geometrically and treated as riding, with C—H = 0.96 Å, O—H = 0.90 Å, and with *U*_iso_(H) = 1.2*U*_eq_(C, O).

### Crystal data

**Table:**

[Zn(C_10_H_6_NO_2_)_2_(H_2_O)]	*F*(000) = 872
*M**_r_* = 427.72	*D*_x_ = 1.753 Mg m^−^^3^
Monoclinic, *C*2/*c*	Mo *K*α radiation, λ = 0.71070 Å
Hall symbol: -C 2yc	Cell parameters from 30 reflections
*a* = 14.929 (2) Å	θ = 3.3–27.5°
*b* = 14.4025 (13) Å	µ = 1.56 mm^−^^1^
*c* = 7.5428 (11) Å	*T* = 293 K
β = 91.961 (6)°	Block, light yellow
*V* = 1620.8 (4) Å^3^	0.30 × 0.30 × 0.20 mm
*Z* = 4	

### Data collection

**Table:**

Rigaku SCXmini diffractometer	1831 independent reflections
Radiation source: fine-focus sealed tube	1741 reflections with *I* > 2σ(*I*)
graphite	*R*_int_ = 0.030
ω scans	θ_max_ = 27.5°, θ_min_ = 3.3°
Absorption correction: multi-scan (*CrystalClear*; Rigaku/MSC, 2005)	*h* = −18→19
*T*_min_ = 0.635, *T*_max_ = 0.732	*k* = −15→18
5552 measured reflections	*l* = −7→9

### Refinement

**Table:**

Refinement on *F*^2^	Primary atom site location: structure-invariant direct methods
Least-squares matrix: full	Secondary atom site location: difference Fourier map
*R*[*F*^2^ > 2σ(*F*^2^)] = 0.026	Hydrogen site location: inferred from neighbouring sites
*wR*(*F*^2^) = 0.069	H-atom parameters constrained
*S* = 1.09	*w* = 1/[σ^2^(*F*_o_^2^) + (0.0375*P*)^2^ + 1.5104*P*] where *P* = (*F*_o_^2^ + 2*F*_c_^2^)/3
1831 reflections	(Δ/σ)_max_ < 0.001
129 parameters	Δρ_max_ = 0.38 e Å^−^^3^
0 restraints	Δρ_min_ = −0.82 e Å^−^^3^

### Special details

**Table:**

Geometry. All e.s.d.'s (except the e.s.d. in the dihedral angle between two l.s. planes) are estimated using the full covariance matrix. The cell e.s.d.'s are taken into account individually in the estimation of e.s.d.'s in distances, angles and torsion angles; correlations between e.s.d.'s in cell parameters are only used when they are defined by crystal symmetry. An approximate (isotropic) treatment of cell e.s.d.'s is used for estimating e.s.d.'s involving l.s. planes.
Refinement. Refinement of *F*^2^ against ALL reflections. The weighted *R*-factor *wR* and goodness of fit *S* are based on *F*^2^, conventional *R*-factors *R* are based on *F*, with *F* set to zero for negative *F*^2^. The threshold expression of *F*^2^ > σ(*F*^2^) is used only for calculating *R*-factors(gt) *etc*. and is not relevant to the choice of reflections for refinement. *R*-factors based on *F*^2^ are statistically about twice as large as those based on *F*, and *R*- factors based on ALL data will be even larger.

### Fractional atomic coordinates and isotropic or equivalent isotropic displacement parameters (Å^2^)

**Table:**

	*x*	*y*	*z*	*U*_iso_*/*U*_eq_	
Zn1	0.5000	0.5000	0.5000	0.01513 (10)	
O1	0.40680 (9)	0.40753 (9)	0.38283 (15)	0.0330 (3)	
O2	0.39589 (8)	0.42662 (9)	0.08731 (15)	0.0325 (3)	
O3	0.5000	0.58494 (9)	0.2500	0.0162 (3)	
N1	0.14620 (8)	0.20508 (9)	0.23221 (16)	0.0219 (3)	
C1	0.22399 (11)	0.18428 (10)	0.3075 (2)	0.0224 (3)	
C2	0.29962 (10)	0.24374 (10)	0.3085 (2)	0.0214 (3)	
C3	0.29358 (10)	0.32726 (10)	0.22272 (18)	0.0179 (3)	
C4	0.19859 (11)	0.43283 (11)	0.0286 (2)	0.0266 (3)	
C5	0.11735 (13)	0.45081 (12)	−0.0528 (2)	0.0342 (4)	
C6	0.04541 (12)	0.38852 (14)	−0.0379 (2)	0.0353 (4)	
C7	0.05608 (11)	0.30808 (13)	0.0555 (2)	0.0293 (3)	
C8	0.13929 (10)	0.28722 (10)	0.14128 (19)	0.0200 (3)	
C9	0.21217 (9)	0.35055 (10)	0.13033 (18)	0.0185 (3)	
C10	0.37322 (9)	0.39320 (10)	0.23183 (19)	0.0193 (3)	
H1	0.2299	0.1255	0.3648	0.027*	
H2	0.3543	0.2252	0.3697	0.026*	
H3	0.2469	0.4754	0.0182	0.032*	
H4	0.1092	0.5063	−0.1217	0.041*	
H5	−0.0115	0.4034	−0.0929	0.043*	
H6	0.0073	0.2655	0.0619	0.035*	
H7	0.5454	0.6264	0.2501	0.021*	

### Atomic displacement parameters (Å^2^)

**Table:**

	*U*^11^	*U*^22^	*U*^33^	*U*^12^	*U*^13^	*U*^23^
Zn1	0.01229 (15)	0.01835 (14)	0.01481 (15)	0.00068 (7)	0.00133 (9)	−0.00354 (7)
O1	0.0360 (7)	0.0416 (7)	0.0210 (6)	−0.0229 (5)	−0.0037 (5)	−0.0001 (5)
O2	0.0303 (6)	0.0465 (7)	0.0211 (5)	−0.0222 (5)	0.0049 (5)	−0.0001 (5)
O3	0.0148 (6)	0.0152 (6)	0.0186 (7)	0.000	0.0000 (5)	0.000
N1	0.0216 (6)	0.0240 (6)	0.0203 (6)	−0.0082 (5)	0.0032 (5)	−0.0022 (5)
C1	0.0273 (8)	0.0196 (6)	0.0205 (7)	−0.0041 (6)	0.0028 (6)	0.0014 (5)
C2	0.0194 (7)	0.0261 (7)	0.0186 (6)	−0.0026 (5)	0.0004 (5)	−0.0005 (5)
C3	0.0182 (7)	0.0212 (6)	0.0144 (6)	−0.0058 (5)	0.0043 (5)	−0.0042 (5)
C4	0.0309 (8)	0.0220 (7)	0.0273 (7)	−0.0018 (6)	0.0061 (6)	0.0004 (6)
C5	0.0382 (10)	0.0338 (8)	0.0309 (9)	0.0100 (7)	0.0044 (7)	0.0077 (7)
C6	0.0255 (8)	0.0503 (10)	0.0299 (9)	0.0083 (7)	−0.0008 (7)	0.0037 (8)
C7	0.0192 (7)	0.0421 (9)	0.0268 (8)	−0.0034 (6)	0.0018 (6)	−0.0010 (7)
C8	0.0185 (7)	0.0250 (7)	0.0167 (6)	−0.0040 (5)	0.0030 (5)	−0.0026 (5)
C9	0.0196 (7)	0.0199 (6)	0.0162 (6)	−0.0029 (5)	0.0039 (5)	−0.0037 (5)
C10	0.0171 (7)	0.0217 (6)	0.0193 (7)	−0.0065 (5)	0.0023 (5)	−0.0032 (5)

### Geometric parameters (Å, °)

**Table:**

Zn1—O2^i^	2.0090 (11)	C2—C3	1.367 (2)
Zn1—O2^ii^	2.0090 (11)	C2—H2	0.96
Zn1—O1^iii^	2.0991 (11)	C3—C9	1.420 (2)
Zn1—O1	2.0991 (11)	C3—C10	1.5213 (19)
Zn1—O3	2.2478 (7)	C4—C5	1.365 (3)
Zn1—O3^iii^	2.2478 (7)	C4—C9	1.422 (2)
O1—C10	1.2459 (18)	C4—H3	0.95
O2—C10	1.2489 (18)	C5—C6	1.407 (3)
O2—Zn1^ii^	2.0090 (11)	C5—H4	0.96
O3—Zn1^ii^	2.2478 (7)	C6—C7	1.362 (3)
O3—H7	0.90	C6—H5	0.96
N1—C1	1.310 (2)	C7—C8	1.413 (2)
N1—C8	1.3695 (19)	C7—H6	0.95
C1—C2	1.417 (2)	C8—C9	1.4244 (19)
C1—H1	0.95		
			
O2^i^—Zn1—O2^ii^	180.0	C1—C2—H2	119.9
O2^i^—Zn1—O1^iii^	92.14 (6)	C2—C3—C9	118.77 (13)
O2^ii^—Zn1—O1^iii^	87.86 (6)	C2—C3—C10	119.28 (13)
O2^i^—Zn1—O1	87.86 (6)	C9—C3—C10	121.94 (13)
O2^ii^—Zn1—O1	92.14 (6)	C5—C4—C9	120.58 (15)
O1^iii^—Zn1—O1	180.0	C5—C4—H3	120.3
O2^i^—Zn1—O3	90.62 (4)	C9—C4—H3	119.1
O2^ii^—Zn1—O3	89.38 (4)	C4—C5—C6	120.78 (16)
O1^iii^—Zn1—O3	89.36 (4)	C4—C5—H4	119.8
O1—Zn1—O3	90.64 (4)	C6—C5—H4	119.4
O2^i^—Zn1—O3^iii^	89.38 (4)	C7—C6—C5	120.50 (16)
O2^ii^—Zn1—O3^iii^	90.62 (4)	C7—C6—H5	120.1
O1^iii^—Zn1—O3^iii^	90.64 (4)	C5—C6—H5	119.4
O1—Zn1—O3^iii^	89.36 (4)	C6—C7—C8	120.20 (15)
O3—Zn1—O3^iii^	180.00 (6)	C6—C7—H6	119.8
C10—O1—Zn1	136.90 (10)	C8—C7—H6	120.0
C10—O2—Zn1^ii^	136.44 (10)	N1—C8—C7	117.58 (13)
Zn1^ii^—O3—Zn1	114.05 (6)	N1—C8—C9	122.53 (13)
Zn1^ii^—O3—H7	109.9	C7—C8—C9	119.90 (14)
Zn1—O3—H7	112.4	C3—C9—C4	124.42 (13)
C1—N1—C8	117.71 (12)	C3—C9—C8	117.57 (13)
N1—C1—C2	123.96 (14)	C4—C9—C8	118.01 (14)
N1—C1—H1	117.8	O1—C10—O2	128.43 (13)
C2—C1—H1	118.2	O1—C10—C3	115.72 (13)
C3—C2—C1	119.31 (14)	O2—C10—C3	115.84 (13)
C3—C2—H2	120.8		

Symmetry codes: (i) *x*, −*y*+1, *z*+1/2; (ii) −*x*+1, *y*, −*z*+1/2; (iii) −*x*+1, −*y*+1, −*z*+1.

### Hydrogen-bond geometry (Å, °)

**Table:**

*D*—H···*A*	*D*—H	H···*A*	*D*···*A*	*D*—H···*A*
O3—H7···N1^iv^	0.90	1.89	2.7920 (15)	174

Symmetry codes: (iv) *x*+1/2, *y*+1/2, *z*.
